# PQSDC: a parallel lossless compressor for quality scores data via sequences partition and run-length prediction mapping

**DOI:** 10.1093/bioinformatics/btae323

**Published:** 2024-05-17

**Authors:** Hui Sun, Yingfeng Zheng, Haonan Xie, Huidong Ma, Cheng Zhong, Meng Yan, Xiaoguang Liu, Gang Wang

**Affiliations:** Nankai-Baidu Joint Laboratory, Parallel and Distributed Software Technology Laboratory, TMCC, SysNet, DISSec, GTIISC, College of Computer Science, Nankai University, Tianjin 300350, China; Nankai-Baidu Joint Laboratory, Parallel and Distributed Software Technology Laboratory, TMCC, SysNet, DISSec, GTIISC, College of Computer Science, Nankai University, Tianjin 300350, China; Institute of Artificial Intelligence, School of Electrical Engineering, Guangxi University, Nanning 530004, China; Nankai-Baidu Joint Laboratory, Parallel and Distributed Software Technology Laboratory, TMCC, SysNet, DISSec, GTIISC, College of Computer Science, Nankai University, Tianjin 300350, China; Key Laboratory of Parallel, Distributed and Intelligent of Guangxi Universities and Colleges, School of Computer, Electronics and Information, Guangxi University, Nanning 530004, China; Nankai-Baidu Joint Laboratory, Parallel and Distributed Software Technology Laboratory, TMCC, SysNet, DISSec, GTIISC, College of Computer Science, Nankai University, Tianjin 300350, China; Nankai-Baidu Joint Laboratory, Parallel and Distributed Software Technology Laboratory, TMCC, SysNet, DISSec, GTIISC, College of Computer Science, Nankai University, Tianjin 300350, China; Nankai-Baidu Joint Laboratory, Parallel and Distributed Software Technology Laboratory, TMCC, SysNet, DISSec, GTIISC, College of Computer Science, Nankai University, Tianjin 300350, China

## Abstract

**Motivation:**

The quality scores data (QSD) account for 70% in compressed FastQ files obtained from the short and long reads sequencing technologies. Designing effective compressors for QSD that counterbalance compression ratio, time cost, and memory consumption is essential in scenarios such as large-scale genomics data sharing and long-term data backup. This study presents a novel parallel lossless QSD-dedicated compression algorithm named PQSDC, which fulfills the above requirements well. PQSDC is based on two core components: a parallel sequences-partition model designed to reduce peak memory consumption and time cost during compression and decompression processes, as well as a parallel four-level run-length prediction mapping model to enhance compression ratio. Besides, the PQSDC algorithm is also designed to be highly concurrent using multicore CPU clusters.

**Results:**

We evaluate PQSDC and four state-of-the-art compression algorithms on 27 real-world datasets, including 61.857 billion QSD characters and 632.908 million QSD sequences. (1) For short reads, compared to baselines, the maximum improvement of PQSDC reaches 7.06% in average compression ratio, and 8.01% in weighted average compression ratio. During compression and decompression, the maximum total time savings of PQSDC are 79.96% and 84.56%, respectively; the maximum average memory savings are 68.34% and 77.63%, respectively. (2) For long reads, the maximum improvement of PQSDC reaches 12.51% and 13.42% in average and weighted average compression ratio, respectively. The maximum total time savings during compression and decompression are 53.51% and 72.53%, respectively; the maximum average memory savings are 19.44% and 17.42%, respectively. (3) Furthermore, PQSDC ranks second in compression robustness among the tested algorithms, indicating that it is less affected by the probability distribution of the QSD collections. Overall, our work provides a promising solution for QSD parallel compression, which balances storage cost, time consumption, and memory occupation primely.

**Availability and implementation:**

The proposed PQSDC compressor can be downloaded from https://github.com/fahaihi/PQSDC.

## 1 Introduction

The development of short reads and long reads sequencing technologies has greatly reduced the cost of obtaining genomics sequencing data, the price dropping from $5292.390/MB in 2002 to $0.006/MB in 2022 (Wetterstrand 2023). This has propelled rapid advancements in virus tracing, precision diagnosis treatment, and new drug development (Hernaez *et al.* 2019, Kredens *et al.* 2020, Liu and Li 2021, Sun *et al.* 2023b). As a result, the growth rate of sequencing data surpasses the Moore’s law (Schaller 1997, Hernaez *et al.* 2019). According to statistics, the China National Gene Bank Sequence Archiving System had stored 14 566 terabytes of genomics sequencing data by April 2024 (Guo *et al.* 2020).

Sequencing data are commonly stored in FastQ files, primarily consisting of descriptive information, sequencing reads, and quality scores data (QSD). Specially, the QSD accounts for approximately 70% in a lossless compressed FastQ file (Chen *et al.* 2022), thus improving the compression performance of QSD is crucial for enhancing the compression ratio and optimizing the storage efficiency. Traditional compressors like 7-Zip (Ipavlov 2010), ZPAQ (Mahoney 2016), and BZIP2 (Seward 2019) perform poorly on compressing QSD files, making them unsuitable for large-scale and long-term sequencing data backup. In recent years, dedicated QSD compressors have been proposed across academic and industry communities, which can be classified as either lossy or lossless depending on whether the decompressed data retains all the original information. A brief review of proposals in the past decade is as follows.

The first category, of QSD lossy compressors, includes QualComp (Ochoa *et al.* 2013), RQS (Yu *et al.* 2014), P/R-Block (Cánovas *et al.* 2014), Quartz (Yu *et al.* 2015), QVZ (Malysa *et al.* 2015), QVZ2 (Hernaez *et al.* 2016), GeneCodeq (Greenfield *et al.* 2016), CROMqs (No *et al.* 2020), (Fu and Dong 2017), Qscomp (lossy) (Voges *et al.* 2018), Crumble (Bonfield *et al.* 2019), ScaleQC (Yu and Yang 2020), and so forth. Those methods overlook the dependence of downstream applications on the initial QSD usage, making the exploration of lossless compressors continue to be a prominent research direction, especially in long-term backup scenarios where data integrity is essential.

The second category, of QSD lossless compressors, includes AQUa (Paridaens *et al.* 2018), LCQS (Fu *et al.* 2020), FCLQC (Cho and No 2021), and so forth. Among them, the AQUa utilizes configurable encoding tools and expands them with a context-adaptive binary arithmetic coding scheme. LCQS compress QSD by maximizing the utilization of hardware resources and consists of four stages: sequences partition, sequences indexing, packing mapping, and parallel compression. By improving the partition and packing mapping rules of LCQS, the CMIC is proposed in Chen *et al.* (2022). FCLQC is a parallel accelerated lossless QSD compressor that achieves lower running times through concurrent programming. Fqzcomp (Bonfield and Mahoney 2013) utilizes a hybrid finite-context model for QSD collections. FastqCLS (Lee and Song 2022) reaches local clustering effects by reordering the original DNA and QSD sequences and then employs the ZPAQ to compress the reordered data streams. The FastQ format files compressors DSRC2 (Roguski and Deorowicz 2014), LWFQZip (Zhang *et al.* 2015), LFQC (Nicolae *et al.* 2015), and LW-FQZip2 (Huang *et al.* 2017) utilize a hybrid encoding strategy [such as run-length and arithmetic coding (Sayood 2017)] to compress QSD streams. Compressors Spring (Chandak *et al.* 2019), Fastore (Roguski *et al*. 2018), ENANO (Dufort y Álvarez *et al.* 2020), GenoZip (Lan *et al.* 2021), RENANO (Dufort y Álvarez *et al.* 2021), and CoLoRd (Kokot *et al.* 2022) employ mechanisms such as QVZ and QSD binning mapping to accomplish both lossy and lossless compression.

By our investigation, existing QSD lossless compressors face the following challenges. (i) The traditional QSD compressors are unsuitable for processing large-scale sequencing data instantly, which restricts their usage in real-time transmission applications. (ii) The high memory consumption of dedicated algorithms restricts their usage on memory-constrained devices, especially when compressing large-scale bio-data. (iii) There is still room for lossless compressors to reduce the storage size of the original data. This work presents a CPU-accelerated lossless compressor for the QSD collections, PQSDC (**P**arallel **QSD C**ompressor), via a parallel sequences-partition model and a four-level run-length prediction mapping model. We analyzed the spatio-temporal complexity of the PQSDC compressor from the perspective of algorithm theory. We also compared PQSDC with state-of-the-art compressors on 27 real-world datasets and validated its superior performance and efficiency.

## 2 Materials and methods

**Figure 1.**
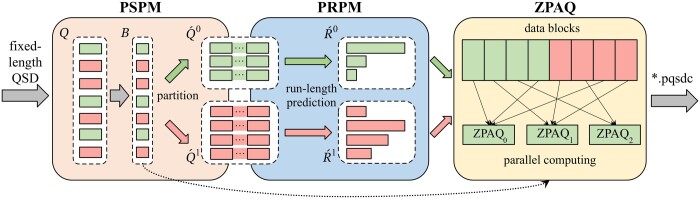
The overall compression workflow of the proposed PQSDC compressor. Examples of parallel PSPM, PRPM, and ZPAQ can be found in the Supplementary Figs S1–S3. The decompression pipeline is the reverse process of the procedure aforementioned.

PQSDC consists of two major components, PSPM and PRPM, and one ZPAQ module, all accelerated for CPU cluster parallelism. Figure 1 shows the compression workflow.

The first component named the Parallel Sequences Partition Model (PSPM), which divides the raw QSD data into two partitions based on the *k*-mer statistics method (Chen *et al.* 2022, Zhong and Sun 2023) and a parallel strategy via data cyclic dividing (Pacheco 2011, Wilt 2013). As Fig. 1 depicts, the PSPM takes *m* fixed-length QSD sequences $Q=\{q_{0},q_{1},\ldots ,q_{m-1}\}$ as input, produces two partitions $\overset{´}{Q}$^0^ and $\overset{´}{Q}$^1^, and a partition marks collection *B* as outputs. By this stage, PQSDC divides raw QSD sequences into relatively small collections and helps to lighten the workload for memory-limited systems. CMIC and LCQS also employ this kind of method. Compared to both schemes, PQSDC reduces the overall memory and time consumption during the partitioning via parallel computing and data-chunk caching techniques (Pacheco 2011, Wilt 2013). Specially, for QSD with variable length, PQSDC activates a preprocessing procedure that adjusts the length of QSD sequences.

The second component called the Parallel Run-length Prediction Mapping Model (PRPM), which maps collections $\overset{´}{Q}$^0^ and $\overset{´}{Q}$^1^ reversibly to shorter sequences and thus reduces the data size. The PRPM model was designed to use the strong correlation between adjacent QSD values, which is also used in previous studies of CMIC and LCQS. Different from both solutions, the PRPM model considers the consecutive and identical characters appearing at diverse mapping levels and applies a modified run-length encoding strategy (Sayood 2017). To decide whether or not to utilize run-length encoding, PRPM apply the multivariate linear regression (Maulud and Abdulazeez 2020) and outputs two collections of mapped sequences namely $\overset{´}{R}$^0^ and $\overset{´}{R}$^1^.

The general-purpose compressor ZPAQ (method-5) utilizes complex context models and arithmetic coding strategy to compress string text, widely used in QSD and DNA reads compression (Fu *et al.* 2020, Chen *et al.* 2022, Lee and Song 2022). To further enhance the compression ratio and speed, as Fig. 1 displays, PQSDC applies a paralleled ZPAQ algorithm running across CPU clusters.

A detailed description of the PSPM and PRPM modes, as well as the paralleled ZPAQ, is presented in the Supplementary Section S1.

## 3 Results and discussion

We utilized seven homogeneous Linux (64-bit CentOS 7.4) servers, each of which was equipped with 2*Intel Xeon Gold 6230 CPU (2.1 Ghz, 40 cores), 192 GB DDR4 SDRAM, and 8*900 GB disk space. PQSDC is compared with four most advanced compressors, namely CMIC (Chen *et al.* 2022), LCQS (Fu *et al.* 2020), Qscomp (Voges *et al.* 2018), and ZPAQ (method-5) (Mahoney 2016). It needs to be addressed that, PQSDC is the first parallel compressor adapted for CPU cluster acceleration. Since we couldn’t find any comparable algorithms, we deliberately designed PQSDC^*a*^, the multicore CPU parallel algorithm, for fairness. PQSDC^*b*^ is the cluster parallel algorithm. The performance of all compressors was evaluated on 27 datasets, including 61.857 billion QSD characters, 632.908 million QSD sequences, and 130.780 GB FastQ files.

We considered various evaluation metrics, including compression ratio (the ratio of compressed file size to the total number of QSD characters) (Dufort y Álvarez *et al.* 2021, Chen *et al.* 2022), compression robustness (the ratio of standard deviation to mean compression ratio multiplied by 100%) (Xing *et al.* 2017), peak memory consumption, time cost, and parallel speedup (Pacheco 2011, Bonfield 2014, Sun *et al.* 2023a, Zhong and Sun 2023). Table 1 shows the overall experimental results. Besides, Supplementary Section S2 provides detailed information about datasets, algorithm descriptions, testing results, and analysis.

**Table 1.** The overall experimental results of PQSDC, ZPAQ, CMIC, LCQS, and Qscomp.

Algorithms	Avg-CR	Wavg-CR	CV	Total-CT	Total-DT	Avg-CPM	Avg-DPM
	(bits/base)	(bits/base)	(%)	(Hours)	(Hours)	(GB)	(GB)
**Short reads:**							
ZPAQ	1.95 (+2.30)	1.02 (+0.69)	**57.97** (−1.72)	7.52 (+69.97)	7.55 (+76.71)	3.46 (+49.34)	3.17 (+49.16)
CMIC	2.05 (+7.06)	1.07 (+5.78)	59.16 (+0.66)	3.41 (+33.83)	3.48 (+49.38)	3.17 (+44.67)	2.91 (+44.66)
LCQS	1.97 (+2.90)	1.03 (+2.23)	82.00 (+28.33)	2.37 (+4.72)	2.26 (+22.17)	5.54 (+68.34)	7.20 (+77.63)
Qscomp	2.04 (+6.38)	1.10 (+8.01)	60.83 (+3.39)	11.27 (+79.96)	11.39 (+84.56)	3.48 (+49.63)	3.17 (+49.16)
PQSDC^*a*^ (Ours)	**1.91** (−0.16)	**1.00** (−0.80)	58.97 (+0.34)	3.58 (+36.81)	2.48 (+29.04)	2.30 (+23.88)	2.12 (+23.87)
PQSDC^*b*^ (Ours)	1.91	1.01	58.77	**2.26**	**1.76**	**1.75**	**1.61**
**Long reads**							
ZPAQ	**3.35** (−0.36)	**3.39** (−0.36)	50.77 (+0.00)	1.93 (+37.89)	2.04 (+64.93)	3.52 (+19.44)	3.15 (+17.42)
CMIC	3.64 (+7.86)	3.71 (+8.18)	51.26 (+0.95)	2.40 (+49.97)	2.41 (+70.29)	3.28 (+13.54)	2.84 (+8.29)
Qscomp	3.84 (+12.51)	3.93 (+13.42)	**49.37** (−2.82)	2.58 (+53.51)	2.61 (+72.53)	3.48 (+18.50)	3.15 (+17.37)
PQSDC^*a*^ (Ours)	3.36 (−0.01)	3.40 (−0.02)	50.80 (+0.07)	4.32 (+72.22)	1.84 (+61.04)	3.82 (+25.63)	2.80 (+7.20)
PQSDC^*b*^ (Ours)	3.36	3.40	50.77	**1.20**	**0.72**	**2.84**	**2.60**

Avg/Wavg-CR, average or weighted average compression ratio (the *weight* for Wavg-CR is the ratio of tested file size to the dataset size); CV, compression robustness performance; Total-CT/DT, total compression or decompression time; Avg-CPM/DPM, average compression or decompression peak memory (a smaller Avg-CR, Wavg-CR, and CV values indicate better compression performance).

Best results are in boldface. Values in parentheses represent the performance gains compared to PQSDC^*b*^, expressed as percentages omitting the “%”. LCQS took over 72 h to compress long reads, thus results are not included. We ensure data integrity by comparing the hash values of the files.

As shown in Table 1, both PQSDC^*a*^ and PQSDC^*b*^ achieved superior overall performance regarding compression ratio and compression robustness. For short reads, compared to baselines, PQSDC^*a*^ gained improvements of 2.46%–7.21% in Avg-CR and 1.47%–8.74% in Wavg-CR; PQSDC^*b*^ improved Avg-CR by 2.30%–7.06% and WAvg-CR by 0.69%–8.01%. For long reads, PQSDC^*b*^ is superior to CMIC and Qscomp in compression ratio, but slightly inferior to ZPAQ by 0.36% only, whether in terms of Avg-CR or Wavg-CR. This is because PQSDC incorporates an additional preprocessing script to record the length of variable-length QSD sequences, introducing extra space overhead. However, compared to ZPAQ, the PQSDC remains highly competitive in memory consumption and time cost. For example, the Total-CT and Total-DT of ZPAQ were 1.61 times and 2.83 times higher than PQSDC^*b*^; the Avg-CPM and Avg-DPM were 1.24 times and 1.21 times, respectively. Furthermore, the CV value of PQSDC^*a*^ and PQSDC^*b*^ ranked only after ZPAQ in short reads and Qscomp in long reads, which indicates that PQSDC is robust when compress QSD with varying data distributions. This is attributed to the proposed PRPM model, which integrates the strengths of mapping and dynamic run-length coding.

In time cost, for short reads, PQSDC^*b*^ saved 69.97%, 33.83%, 4.72%, 79.96%, and 36.81% of Total-CT, as well as 76.71%, 49.38%, 22.17%, and 84.56% of Total-DT, compared to ZPAQ, CMIC, LCQS, and Qscomp, respectively. Additionally, PQSDC^*a*^ also demonstrated superior time consumption in both compression and decompression processes. The Total-CT outperformed ZPAQ and Qscomp, while the Total-DT was better than ZPAQ, CMIC, and Qscomp. For long reads, compared to ZPAQ, CMIC, and QScomp, PQSDC^*a*^ had the worst Total-CT but the best Total-DT. However, with CPU-cluster acceleration, PQSDC^*b*^ achieved time savings of 37.89%–72.22% in TotalCT and 61.04%–72.53% in Total-DT compared to tested algorithms.

Besides, PQSDC is memory-friendly. Taking PQSDC^*b*^ as an example, for short reads, it saved 44.67%–68.34% of Avg-CPM in compression and 44.66%–77.63% of Avg-DPM in decompression. For long reads, PQSDC^*b*^ saved 13.54%–25.63% Avg-CPM and 7.02%–17.42% Avg-DPM, respectively. The remarkable performance advantages of PQSDC in processing memory consumption are mainly attributed to the designed PSPM model, which is based on *k*-mer statistics, memory optimization, and multicore CPU parallel.

## 4 Conclusion

In this study, we propose a parallel QSD compressor, named PQSDC, which achieves the storage-time-memory balance. The main techniques are two parallel optimization models, namely PSPM and PRPM. We evaluate the compression performance and robustness of PQSDC and benchmark algorithms on 27 datasets. The practical results demonstrate that PQSDC achieves competitive advantages in terms of time cost, memory consumption, and compression robustness, with high compression ratio.

With the development of third-generation and fourth-generation high-throughput genomic sequencing technologies, genomic sequencing data are characterized by high error rates, long lengths, large sizes, and diverse species origins. By integrating our current work, future research directions are as follows. (i) Explore how to further optimize the compression ratio by utilizing the data redundancy between different sequencing files without significantly increasing memory overhead. (ii) Design an efficient CPU-GPU hybrid parallel algorithm based on PQSDC to run on GPU clusters. (iii) Develop a parallel framework for FastQ files compression based on PQSDC, which combines existing DNA sequence compression algorithms with memory, I/O, and SIMD (Single Instruction Multiple Data) optimization methods.

## Supplementary Material

btae323_Supplementary_Data

## Data Availability

The datasets generated and analyzed during the current study are available in the PQSDC repository (https://github.com/fahaihi/PQSDC).
