# Supplementary material for: PQSDC: a parallel lossless compressor for quality scores data via sequences partition and run-length prediction mapping
Source: Bioinformatics. 2024 May 17;40(5):btae323. doi: 10.1093/bioinformatics/btae323 (PMC11139522; doi:10.1093/bioinformatics/btae323)
Supplement: btae323_Supplementary_Data [file btae323_supplementary_data.pdf]

PQSDC: A Parallel Lossless Compressor for Quality Scores Data via Sequences Partition and Run-Length Prediction Mapping (Supplementary Materials)

Hui Sun, Yingfeng Zheng, Haonan Xie, Huidong Ma, Cheng Zhong,
Meng Yan, Xiaoguang Liu, and Gang Wang

May 15, 2024

This document provides implementation details of the PQSDC compressor, algorithm description analysis, and additional experimental results. Our compression tool PQSDC is available at `https://github.com/fahaihi/PQSDC`.

Contents

1 Details of PQSDC Compressor **2**
- 1.1 Overview . . . 2
- 1.2 Parallel Sequences Partition Model . . . 2
- 1.3 Parallel Run-length Prediction Mapping Model . . . 3
  - 1.3.1 First-Level Mapping . . . 4
  - 1.3.2 Second-Level Mapping . . . 5
  - 1.3.3 Third-Level Mapping . . . 5
  - 1.3.4 Fourth-Level Mapping . . . 5
  - 1.3.5 Case Study of PRPM Model . . . 6
- 1.4 Final Compression Using ZPAQ Algorithm . . . 7
- 1.5 Algorithm Description and Analysis . . . 8

2 Supplementary Results and Analysis **10**
- 2.1 Experimental Datasets . . . 10
- 2.2 Benchmark Algorithms and Parameter Configurations . . . 11
- 2.3 Evaluation Metrics . . . 12
- 2.4 Compression Performance . . . 12
  - 2.4.1 Compression Ratio and Robustness . . . 12
  - 2.4.2 Time Consumption . . . 14
  - 2.4.3 Peak Memory Consumption . . . 15
- 2.5 Algorithm Ablation Experiments . . . 16
- 2.6 Parallel Performance . . . 16
  - 2.6.1 Multi-core CPU Parallel Acceleration . . . 16
  - 2.6.2 Multi-node CPU Cluster Parallel Acceleration . . . 17

1 Details of PQSDC Compressor

In this section, we first give an overview of our proposed PQSDC compressor, then detail the parallel sequences partition model and the parallel run-length prediction mapping model used by PQSDC. Secondly, we describe how PQSDC achieves joint compression and decompression by using the multi-cores CPU cluster. Finally, we provide the description of the PQSDC algorithm and analyze its algorithmic complexity.

1.1 Overview

Our proposed PQSDC compressor consists of two major data processing components and a series of data encoding scripts. The first component is the Parallel Sequences Partition Model (PSPM), which partitions the raw compressed data into two partitions based on the k-mer feature statistics technology. The PSPM model takes m fixed-length QSD sequences $Q = \{q_0, q_1, ..., q_{m-1}\}$ as input, produces two partitions $\acute{Q}^0$ and $\acute{Q}^1$, and a partition marks collection B as major outputs. The second component is the Parallel Run-length Prediction Mapping Model (PRPM), which reversibly maps the original QSD characters and reduces the data size of partition collections $\acute{Q}^0$ and $\acute{Q}^1$. PRPM outputs two collections of mapped sequences, namely $\acute{R}^0$ and $\acute{R}^1$.

In order to further enhance the compression ratio and speed, PQSDC applies the CPU clusters accelerated general-purpose compression algorithm ZPAQ [1] to the mapped data streams. After that, it packages all compressed data streams into a single file "*.pqsdc", which is the final output. For variable-length ultra-long QSD datasets, PQSDC offers the "$-l$" parameter in the command line, which activates an preprocessing encoding script that corrects the QSD sequence length.

1.2 Parallel Sequences Partition Model

In the parallel sequence partition stage, we aim to divide raw QSD sequences into relatively small-sized collections, which helps to lighten the workload for memory-limited systems. To this end, a simple approach which performs block-level partition on the QSD sequences might be helpful. However, this ordinary method overlooks the redundant information between different QSD sequences, which can be leveraged to improve the compression ratio during the mapping stage.

In order to leverage the redundancy properties in different QSD sequences, we present a parallel sequences partition model based on the k-mer feature statistics method[2, 3, 4] and the data cycle-parallel strategy [5, 6]. Different from the partition solutions in CMIC[2] and LCQS[3] compressors, the PQSDC algorithm reduces the overall partition memory and time consumption via parallel computing and data chunk caching techniques [5, 6]. Fig 1 presents an illustrative diagram of the PSPM model performing binary partition.

In Fig 1, $Q = \{q_0, q_1, ...q_i, ..., q_{m-1}\}$, $q_i = \{q_{i,0}, q_{i,1}, ...q_{i,j}, ..., q_{i,n-1}\}$, $q_{i,j} \in \{\Theta\}$ denotes the j-th QSD string character, Θ denotes the QSD alphabet, and n denotes the fixed QSD length. $B = \{b_0, b_1, ..., b_i, ..., b_{m-1}\}$ is used to record partition results for QSD sequences collection Q, where $0 \leq i \leq m-1$ and $b_i \in \{0, 1\}$. Given a sequence q_i, the corresponding b_i is computed according to Eq (1). In order to accelerate the computation of collection B, the PQSDC compressor leverages Pr CPU cores to execute the PSPM model.

$$b_i^p = \begin{cases} 1, & \text{if } (\frac{\sum_{j=0}^{n-k+1} S(q_{i,j:j+k-1}^p)}{NFactor} \geq \alpha) \\ 0, & \text{otherwise} \end{cases} \quad (1)$$

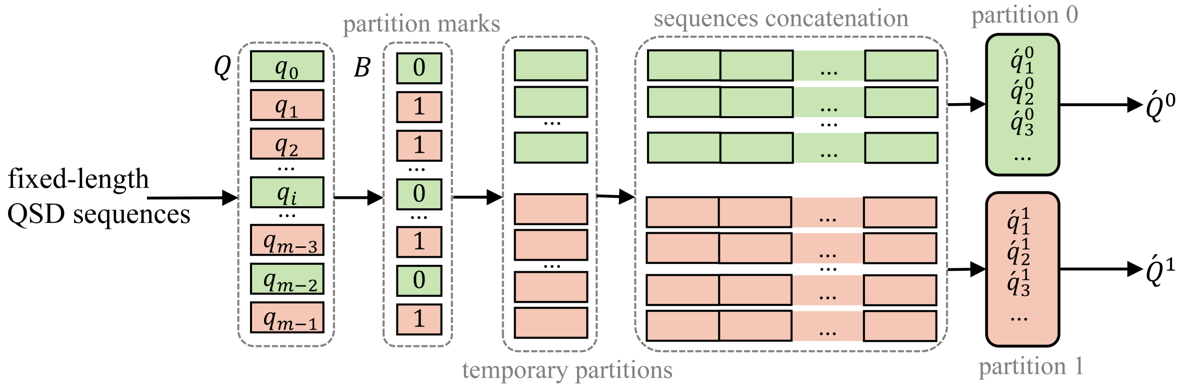

Figure 1: **A QSD sequences binary partition example of the PSPM model**

In Eq (1), k denotes the length of the sliding window for k-mer splitting operations, α denotes the partition threshold factor, and the parallel parameter $p = \lfloor \frac{i}{Pr} \rfloor$ denotes the p-th CPU core. The function $S(q^p_{i,j:j+k-1})$ represents the k-mer statistical value collected at the j-th position of the string q_i, which is shown in Eq (2). $NFactor$ is the sampling normalization factor, and its calculation is shown in Eq (3). Here, we have $i = 0, 1, ..., m-1$ and $j = 0, 1, ..., n-k+1$.

$$S(q^p_{i,j:j+k-1}) = \frac{\sum_{\acute{i}=0}^{M-1}(\sum_{\acute{j}=0}^{n-k+1} I(q^p_{i,j:j+k-1} = q_{\acute{i},\acute{j}:\acute{j}+k-1}))}{(n-k+1)\times M} \tag{2}$$

$$NFactor = max\{\sum_{\acute{j}=0}^{n-k+1} S(q_{\acute{i},\acute{j}:\acute{j}+k-1})\}, \acute{i} = 0, 1, ..., M-1. \tag{3}$$

In Eq (2), M denotes the number of random sampling from collection Q. The expression $I(q^p_{i,j:j+k-1} = q_{\acute{i},\acute{j}:\acute{j}+k-1})$ denotes an indicator function, which takes value $\{0, 1\}$. For example, $I($"ACJJK" = "ACJJK"$) = 1$, and $I($"ACJJK" = "YCJJK"$) = 0$. In Eq (3), the purpose of the sampling normalization factor $NFactor$ is to scale the k-mer statistical score values of the QSD sequences to the range [0,1]. Eq (3) simplifies the selection of the partition threshold factor α in Eq (1) and facilitates extending the sequences binary classification partition model to multiple categories.

Given the partition results $B = \{b_0, b_1, ..., b_i, ..., b_{m-1}\}$ via Eq (1-3), a sequences concatenation operation is performed according to the user pre-defined parameter N, which balances the calculation loads among different cores of CPU during the subsequent mapping stage. After that, the PQSDC obtains the QSD partitions $\acute{Q}^v = \{\acute{q}^v_0, \acute{q}^v_1, ..., \acute{q}^v_{\lceil \frac{B^v}{N} \rceil - 1}\}$, where $B^v = \sum_{i=0}^{m-1} I(b_i = v)$ and $v = 0, 1$. For the partitioned QSD sequence sets $\acute{Q}^v$ $(v = 0, 1)$, PQSDC applies a four-stage parallel run-length prediction mapping model and further reduces the data size to be compressed, detailed in the next section.

1.3 Parallel Run-length Prediction Mapping Model

Previous study [7] showed that adjacent quality score values indicate a strong correlation. To leverage such data characteristic, inspired by the QSD mapping concept in [2] and [3], we propose a new parallel run-length prediction mapping model, which aims to minimize the data size to be compressed and reduce the time and peak memory consumption. Different from the mapping solutions of CMIC [2] and LCQS [3], the PRPM model considers the consecutive and identical characters appearing at diverse mapping levels and applies modified run-length encoding strategy[8]. To decide whether or not to utilize run-length

encoding, we apply the multivariate linear regression algorithm [9].

Before providing a detailed explanation of the four-stage PRPM model, we will present some modeling notations. Collection $\acute{B}^v = \{\acute{b}_0^v, \acute{b}_1^v, ..., \acute{b}_i^v, ..., \acute{b}_{\lceil \frac{B^v}{N} \rceil - 1}^v\}$ denotes the mapping marks for QSD sequences set $\acute{Q}^v$, which is a two-dimensional array. Collection $\acute{b}_i^v = \{\acute{b}_{i,0}^v, \acute{b}_{i,1}^v, ..., \acute{b}_{i,j}^v\ \acute{b}_{i,n-1}^v\}$ records the mapping marks for sequence $\acute{q}_i^v \in \acute{Q}^v$, where $\acute{b}_{i,j}^v \in \mathbb{Z}$, $0 \leq \acute{b}_{i,j}^v \leq 255$, $v = 0, 1$, $i = 0, 1, ..., \lceil \frac{B^v}{N} \rceil - 1$, and $j = 0, 1, ..., N \times n - 1$. Given QSD string $\acute{q}_i^v$, symbol C_i^v denotes the mode character (the character that appears most often) and $E_i^v = \{e_{i,j}^v\}$ represents the run-length marker collection. As an example, if sequence $\acute{q}_0^0$ takes "GMFFFWCCCCO", then $E_0^0 = \{1, 1, \underline{3}, 1, 1, 1, \underline{4}, 1, 1, 1, 1, 1\}$ and $C_0^0 =$ "C".

The PRPM mapping model aims to reduce redundancy in the sequence $\acute{q}_i^v$ by utilizing the mode character C_i^v. It divides the sequence into four mapping intervals based on the mode character and dynamically uses the run-length encoding strategy [8] to reduce repeated QSD characters within each interval. The PRPM model involves an amount of computationally-intensive mapping operations. Thus, the PQSDC also utilizes a data cycle-parallel strategy to accelerate the mapping computation [4, 6].

1.3.1 First-Level Mapping

The first-level mapping stage maps continuous mode characters to value space [201,255] using the run-length encoding method, where the length of continuous mode characters less than 54 (255-201). Current run-length encoding techniques can effectively eliminate consecutive repeated characters in the QSD strings and thus compress the data size. However, the actual compression gain depends on the frequency of occurrence of consecutive repeated characters; thus, it is unstable. In addition, run-length encoding may increase the information entropy and be unfavorable for the subsequent arithmetic coding compression[8, 10]. To solve these problems, the PRPM model introduces a switch factor β, which determines whether or not to apply the run-length encoding. The PQSDC parallel updates the mapping marks collection $\acute{B}^v = \{\acute{b}_i^{p,v}\}$ and the local index parameter j using the PRPM first-level mapping model, as shown in Eq (4).

$$\acute{b}_{i,j}^{p,v}; j = \Big\{ 201 + e_{i,j}^{p,v}; j + e_{i,j}^{p,v}, if((\acute{q}_{i,j}^{p,v} = C_i^{p,v}) \wedge (e_{i,j}^{p,v} \geq 3) \wedge (y_i^{p,v} \geq \beta)) \tag{4}$$

In Eq (4), the notation $\acute{q}_{i,j}^{p,v}$ represents the j-th QSD character in i-th QSD string appeared at v-th sequences collection $\acute{Q}^v$. The parameter $y_i^{p,v} \in [0, 1]$ represents the predicted maximum compression gain of sequence $\acute{q}_i^{p,v}$. When the condition $y_i^{p,v} \geq \beta$ is satisfied, the PRPM model applies the run-length encoding to the corresponding mode characters whose length is no less than 3. Here, we named β as a dynamic adjustment switch factor (because it determines whether PRPM performs run-length encoding), $i = 0, 1, ..., \lceil \frac{B^v}{N} \rceil - 1$, $j = 0, 1, ..., N \times n - 1$, $p = \lfloor \frac{i}{Pr} \rfloor$, and $v = 0, 1$. In our solution, the PQSDC adopts a multivariate linear regression algorithm [9] to predict the local parameter $y_i^{p,v}$, and its computation is shown in Eq (5).

$$y_i^{p,v} = w_0 + \sum_{t=0}^{3} w_{t+1} \times x_{i,t}^{p,v} \tag{5}$$

In Eq (5), $x_{i,t}^{p,v}$ $(t = 0, 1, 2, 3)$ denotes the features of string $\acute{q}_i^{p,v}$, where $x_{i,0}^{p,v} = \frac{C_i^{p,v}}{n \times N}$ denotes the proportion of the mode characters, $x_{i,1}^{p,v} = \frac{\sum_{j=0}^{n \times N - 1} I((\acute{q}_{i,j}^{p,v} - \acute{q}_{i,j-1}^{p,v})>7}{n \times N - 1}$ denotes the proportion of characters with adjacent quality values greater than 7, $x_{i,2}^{p,v} = \frac{\sum_{j=0}^{n \times N - 1} (e_{i,j}^{p,v} > 3)}{n \times N}$ denotes the proportion of characters with a run-length greater than 3, and $x_{i,3}^{p,v} = \frac{Sor(\acute{q}_i^{p,v})}{|\Theta|}$ denotes the number of different characters of $\acute{q}_i^{p,v}$

divide the QSD alphabet size. In Eq (5), w_0 and w_{t+1} are the modeling weight parameters. We fitted the weight parameters w_0 and w_{t+1}, as well as the dynamic adjustment switch factor β (used in Eq (5)) through an offline learning method on real-world QSD sequencing datasets, where $t = 0, 1, 2, 3$. The detailed configuration of these parameters can be found in the experimental section.

1.3.2 Second-Level Mapping

The purpose of the PRPM second-level mapping model is to map the QSD characters nearest the mode character C_i^v to the intervals [137,200] and [201,255]. This stage also adopts the run-length switching factor shown in Eq (5) for dynamic adjustment to achieve the best mapping consequence. Eq (6) shows our second-level mapping rule.

$$\acute{b}_{i,j}^{p,v}; j = \begin{cases} 201 + e_{i,j}^{p,v}; j + e_{i,j}^{p,v}, \\ if((C_i^{p,v} > \acute{q}_{i,j}^{p,v} \geq (C_i^{p,v} - 3)) \wedge (e_{i,j}^{p,v} > 6) \wedge (y_i^{p,v} \geq \beta)) \\ (\acute{q}_{i,j}^{p,v} - C_i^{p,v} + 3) \times 2^4 + (\acute{q}_{i,j+1}^{p,v} - C_i^{p,v} + 3) \times 2^2 + (\acute{q}_{i,j+2}^{p,v} - C_i^{p,v} + 3) + 137; j + 3, \\ if(C_i^{p,v} \geq \acute{q}_{i,j}^{p,v}, \acute{q}_{i,j+1}^{p,v}, \acute{q}_{i,j+2}^{p,v} \geq (C_i^{p,v} - 3)) \end{cases} \tag{6}$$

In Eq (6), the PRPM model employs the run-length encoding technique to those QSD characters that satisfy $C_i^{p,v} > \acute{q}_{i,j}^{p,v} \geq (C_i^{p,v} - 3)$. If the length of consecutive repeated characters exceeds 6 ($e_{i,j}^{p,v} > 6$) and meets the condition $y_i^{p,v} \geq \beta$, the PRPM model maps them to the interval [201, 255]. Otherwise, PRPM adopts a mapping strategy similar to LCQS [3] and maps three QSD characters $\acute{q}_{i,j}^{p,v}$,$\acute{q}_{i,j+1}^{p,v}$, and $\acute{q}_{i,j+2}^{p,v}$ close to $C_i^{p,v}$ to the interval [137, 200]. Through the PRPM second-level dynamic run-length mapping, PQSDC compresses three consecutive characters closest to the mode characters into a single character in the worst cases.

1.3.3 Third-Level Mapping

This stage packages the continuous QSD characters close to C_i^v to [73,136] and [201,255] spaces. The proposed PRPM third-level mapping model is shown in Eq (7).

$$\acute{b}_{i,j}^{p,v}; j = \begin{cases} 201 + e_{i,j}^{p,v}; j + e_{i,j}^{p,v}, \\ if(((C_i^{p,v} - 3) > \acute{q}_{i,j}^{p,v} \geq (c_i^{p,v} - 7)) \wedge (e_{i,j}^{p,v} > 9) \wedge (y_i^{p,v} \geq \beta)) \\ (\acute{q}_{i,j}^{p,v} - C_i^{p,v} + 7) \times 2^3 + (\acute{q}_{i,j+1}^{p,v} - C_i^{p,v} + 7) + 73; j + 2, \\ if(C_i^{p,v} \geq \acute{q}_{i,j}^{p,v}, \acute{q}_{i,j+1}^{p,v} \geq (C_i^{p,v} - 7)) \end{cases} \tag{7}$$

In Eq (7), the PRPM model first employs the run-length encoding technique to those QSD characters that satisfy $(C_i^{p,v} - 3) > \acute{q}_{i,j}^{p,v} \geq (c_i^{p,v} - 7)$. For character $\acute{q}_{i,j}^{p,v}$, if its repeat length is greater than 9 ($e_{i,j}^{p,v} > 9$), PRPM mapping them to the interval [201, 255]. If the run-length encoding is not applicable, PRPM maps two consecutive characters $\acute{q}_{i,j}^{p,v}$ and $\acute{q}_{i,j+1}^{p,v}$ to the interval [73,136]. In the worst-case scenario, the PRPM model maps two QSD characters into one character. This compact representation of characters effectively saves storage space for storing QSD sequences.

1.3.4 Fourth-Level Mapping

After completing the aforementioned three stages of mapping, PRPM utilizes a fourth-level strategy to handle cases where QSD values deviate from the predefined rules. This strategy maps the original QSD characters to the intervals [0, 72] and [201, 255]. The specific approach for the fourth-level mapping is

defined in Eq (8).

$$\acute{b}_{i,j}^{p,v};j=\begin{cases}201+e_{i,j}^{p,v};j+e_{i,j}^{p,v},\\ if(((C_i^{p,v}-7>\acute{q}_{i,j}^{p,v})\vee(\acute{q}_{i,j}^{p,v}>C_i^{p,v}))\wedge(e_{i,j}^{p,v}>3)\wedge(y_i^{p,v}\geq\beta))\\ \acute{q}_{i,j}^{p,v}-32;j+1,\\ if((C_i^{p,v}-7>\acute{q}_{i,j}^{p,v})\vee(\acute{q}_{i,j}^{p,v}>C_i^{p,v}))\end{cases} \tag{8}$$

In Eq (8), the PRPM model utilizes the run-length encoding strategy to those QSD characters that meet $C_i^{p,v}-7>\acute{q}_{i,j}^{p,v}$ or $\acute{q}_{i,j}^{p,v}>C_i^{p,v}$. If the length of consecutive repeated characters exceeds 3 ($e_{i,j}^{p,v}>3$), the PRPM model maps them to the interval [201, 255]. Otherwise, the PRPM model maps characters $\acute{q}_{i,j}^{p,v}$ to the interval [0, 72]. Therefore, in the best cases, the PRPM model encodes QSD characters with a run length greater than 3 into a single character space. In the worst cases, the PRPM performs a one-to-one character mapping rule.

1.3.5 Case Study of PRPM Model

The PQSDC employs a reversible numerical mapping method, PRPM, which achieves lossless compression of QSD sequences by mapping the original data to a smaller coding range.

Let $\acute{R}^v=\{\acute{r}_0^v,\acute{r}_1^v,...,\acute{r}_i^v,...,\acute{r}^v_{\lceil\frac{B^v}{N}\rceil-1}\}$ represent the mapping results collection for QSD sequences, where $v=0,1$. To better explain the PRPM model, Fig 2 provides a PQSDC mapping case study, which utilizes the PRPM model to eliminate QSD redundancy information.

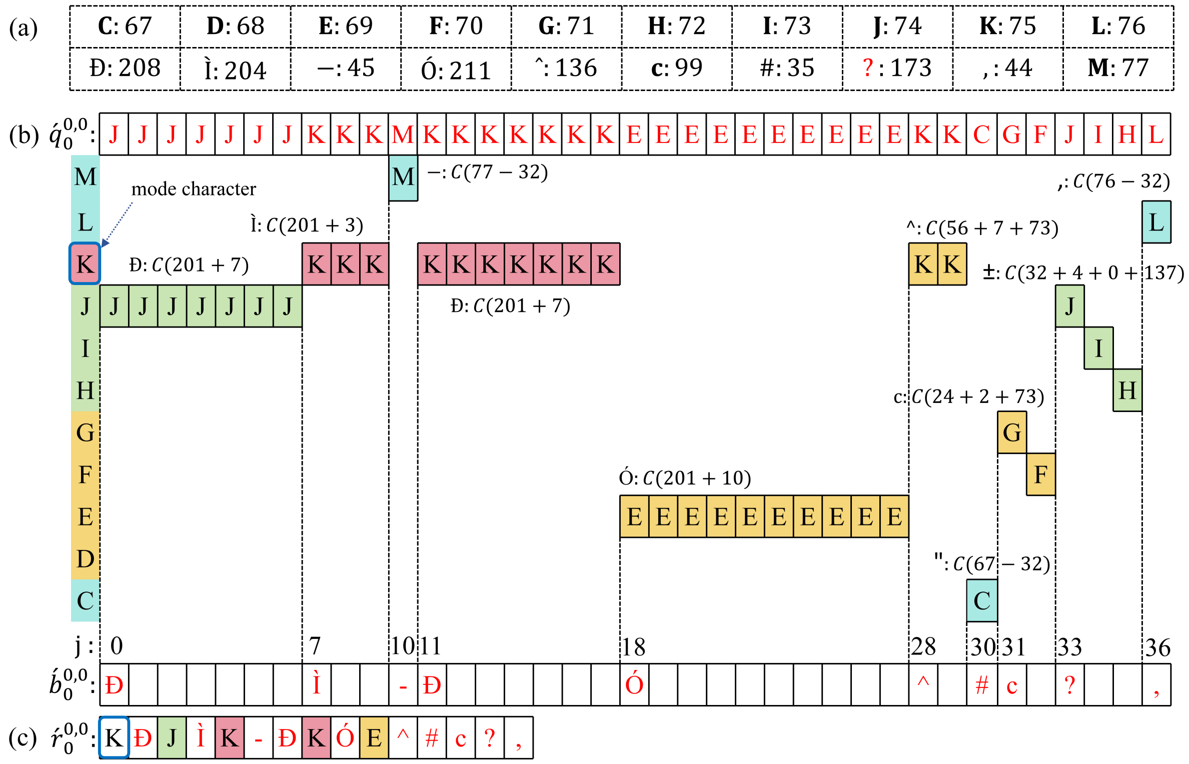

Figure 2: **An example of mapping sequence $\acute{q}_0^{0,0}$ to $\acute{r}_0^{0,0}$ using PRPM four-level mapping model in our PQSDC compressor.** (a) The QSD "Character-ASCII" relationship. (b) A demonstration example of calculating mapped results record set $\acute{b}_0^{0,0}$. (c) PRPM final mapped results $\acute{r}_0^{0,0}$.

Fig 2(a) illustrates the QSD "Character-ASCII" relationship. For instance, "D:68" indicates that the

ASCII value of character “D” is 68. In other word, $ASCII$(D) = “68” and $Char$(68) = “D”. In Fig 2(b), the sequence length of $\acute{q}_0^{0,0}$ is 37 and the mode character is “K”. The 0-th CPU core performs the PRPM model for the sequence $\acute{q}_0^{0,0}$, which is the 0-th string in the collection $\acute{Q}^0$. The PRPM model employs four mapping rules as shown in Eq (4-8), and the specific update process is as follows.

Firstly, at the first-level mapping stage (characters with a red background in the diagram), the PRPM model maps the three consecutive characters “KKK” at $j = 7$ to “Ì”, and maps the seven consecutive mode characters “KKKKKKK” at $j = 11$ to “Đ” using Eq (4). Secondly, at the second-level mapping stage (as shown in the background of green), the PRPM model maps continue QSD characters “JJJJJJJ” at $j = 0$ to “Đ” using run-length encoding strategy. For characters “JIH” at $j = 33$, the PRPM model maps them to character “?” using preset mapping rules as shown in Eq (6). Thirdly, at the third-level mapping stage (as shown background of orange), the PRPM model maps string “EEEEEEEEEE” begin with $j = 18$ to character “Ó”, and “GF” at $j = 31$ to “c” using Eq (7). At this stage, for adjacent QSD values “KK” at $j = 28$, the PRPM model maps them to a single character “^”. Finally, at the fourth-level mapping stage (as shown in the background of blue), the PRPM model applies char-to-char mapping using Eq (8). More specifically, it maps “M” to “-”, “C” to “#”, and “L” to “,” at $j = 10$, $j = 30$, and $j = 36$, respectively.

After the four mapping stages, in Fig 2(c), the PRPM gets the final mapped QSD sequence $\acute{r}_0^{0,0} =$ “KĐJÌK $-$ ĐKÓE $\wedge$ #c?, ”, which length is 15. Compared with $\acute{q}_0^{0,0}$, the proposed PRPM mapping model reduces the data size up to $\frac{37-15}{37} \times 100\% = 59.459\%$. The PQSDC compressor utilizes the PRPM model to encode redundant characters information in QSD. Due to the linear mapping rules employed by the PRPM model, it is possible to losslessly recover string $\acute{q}_0^{0,0}$ from string $\acute{r}_0^{0,0}$. For example, for character “#” at position 30 ($j = 30$) in $\acute{b}_{0,j}^{0,0}$, the PRPM model restore character “C” by performs $Char$(35+32)=“C" calculation.

1.4 Final Compression Using ZPAQ Algorithm

After the aforementioned parallel sequences partition and parallel run-length prediction mapping stages, PQSDC losslessly restores PSPM partitions $\acute{Q}^v$ using the mapping results collections $\acute{B}^v$ and final mapped sequence collection $\acute{R}^v$. Because the key information of set $\acute{B}^v$ is preserved in collection $\acute{R}^v$, the decompression process of PQSDC can reconstruct set $\acute{B}^v$ through inverse mapping. Therefore, PQSDC only needs to store the mapping collection files $\acute{R}^0$ and $\acute{R}^1$ generated by the PRPM model, along with the partition marks collection file B generated by the PSPM model, where $v = 0, 1$.

The algorithm ZPAQ (method-5) [1] utilizes a complex high-order context mixture model and arithmetic coding strategy to compress string text, widely used in QSD and DNA reads compression [2, 3, 11]. In order to reduce storage space of files generated by PRPM and PSPM models, the PQSDC compresses those collection files through the ZPAQ algorithm and generate the final compressed file “$*.pqsdc$”. Due to the high time cost of cascading ZPAQ for final compression, PQSDC assigns $\acute{R}^v$ to the multi-core CPU clusters through the data block-cycle-parallel strategy [4, 6], where $v = 0, 1$.

Fig 3 shows an example of PQSDC utilizing three CPU cluster nodes to parallel accelerate the compression of the ZPAQ algorithm.

In Fig 3, PQSDC divides collection files $\acute{R}^0$ and $\acute{R}^1$ into four equal blocks, which are periodically assigned to three CPU nodes (each cluster node runs a CPU multi-core accelerated ZPAQ compressor) for parallel computation. In the application scenarios of timely data backup for large-scale sequencing databases, the compression server system often consists of a cluster array of multiple computing-limited CPU nodes. PQSDC is the first dedicated parallel algorithm that explores using distributed computing resources for QSD compression. Due to its highly flexible parameter settings, PQSDC also supports parallel accelerated computation on single node with multiple CPU cores. In the experimental section,

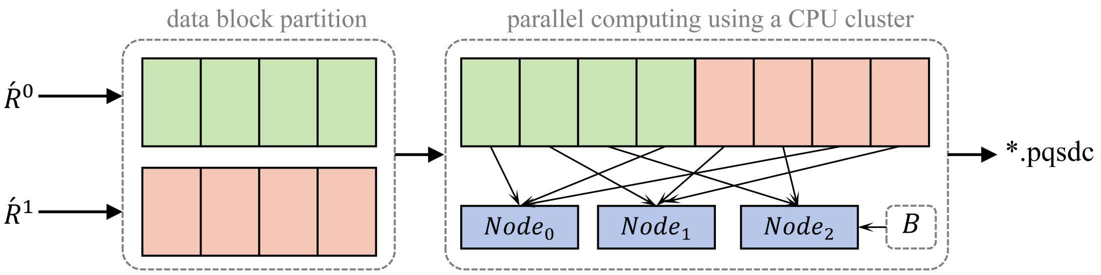

Figure 3: **PQSDC employs three CPU cluster nodes to accelerate the computation process of the ZPAQ algorithm.**

we will reveal more details of these parallelism settings and their implementation for accelerated lossless QSD compression.

1.5 Algorithm Description and Analysis

Let $Bl = \{bl_0, bl_1, ..., bl_{2\times G-1}\}$ denotes the data collection obtained by the PQSDC compressor through the average partition on data stream $\acute{R}^v$ using the user's preset parallel block parameter G. The parameter Nu represents the number of cluster nodes. Algorithm 1 formally expresses the multi-cores CPU cluster based parallel lossless quality scores data compression algorithm PQSDC.

Algorithm 1 PQSDC

Input : the fixed-length QSD sequences collection $Q = \{q_0, q_1, ..., q_{m-1}\}$; the length of fixed QSD sequences n; the number of fixed-length QSD sequences m; the number of random sampling M; the sliding window parameter k for k-mer operations; the partition threshold factor α; the dynamic adjustment switch factor β; the sequences concatenation parameter N; the weights $\{w_0, w_1, w_2, w_3, w_4\}$; the number of CPU cores Pr; the parallel block parameter G; the number of cluster nodes Nu.
Output : the compressed output file $*.pqsdc$.

BEGIN.
1: Parallel Initialization $B \leftarrow \{b_0, b_1, ..., b_{m-1}\}$, $b_i \leftarrow 0$, where $i = 0, 1, ..., m-1$;
2: Initialize the k-mer frequency table $F \leftarrow \{\text{‰}\}$;
3: Initialize $NFactor \leftarrow$"$-INF$"; /* *-INF* represents negative infinity. */

Function : QSD sequences partition.
4: Parallel traversal of the first M strings in collection Q, counting k-mers using parameter k and populating the frequency table F;
5: **for** $p = 0$ to $Pr - 1$ **do-para** /* Pallel compute the normalization factor *NFactor* based on Eq (2) and Eq (3). */
6: **for** $i = 0$ to $M - 1$ **do-para**
7: **if** $p = \lceil \frac{i}{Pr} \rceil$ **and** $\frac{\sum_{j=0}^{n-k+1} F[q^p_{i,j:j+k-1}]}{(n-k+1)\times M} > NFactor$ **then**
8: $NFactor \leftarrow \frac{\sum_{j=0}^{n-k+1} F[q^p_{i,j:j+k-1}]}{(n-k+1)\times M}$; /* Mutually update *NFactor* */
9: **end if**(line-7)
10: **end for**(line-6)
11: **end for**(line-5)

12: **for** $p = 0$ to $Pr - 1$ **do-para** /* Parallel updates B via Eq (1). */
13: **for** $i = 0$ to $m - 1$ **do-para**
14: **if** $p = \lceil \frac{i}{Pr} \rceil$**and** $\frac{\sum_{j=0}^{n-k+1} F[q_{i,j:j+k-1}^{p}]}{NFactor \times (n-k+1) \times M} \geq \alpha$ **then**
15: $b_i^p \leftarrow 1$;
16: **else** $b_i^p \leftarrow 0$;
17: **end if**(line-14)
18: **end for**(line-13)
19: **end for**(line-12)

Function : QSD sequences concatenation.

20: Initialize collections $\acute{Q}^v \leftarrow \{\text{‰}\}, where\ v = 0, 1$;
21: Perform parallel concatenation operation on Q based on parameter N and collection B, as well as record the result in collection $\acute{Q}^v$, where $v = 0, 1$;
22: Parallel initialization the mapping sets $\acute{B}^v \leftarrow \{\acute{b}_i^v\}$, where $\acute{b}_i^v \leftarrow \{\acute{b}_{i,j}^v \leftarrow \text{“}NUL\text{”}\}$, $i = 0, 1, ..., \lceil \frac{B_v}{N} \rceil - 1$, $j = 0, 1, ..., N \times n - 1$, $v = 0, 1$;

Function : QSD run-length prediction mapping.

23: **for** $v = 0$ to 1 **do** /* Iterate over the sets $\acute{Q}^0$ and $\acute{Q}^1$ */
24: **for** $p = 0$ to $Pr - 1$ **do-para** /* Execute the PRPM model */
25: **for** $i = 0$ to $\lceil \frac{B_v}{N} \rceil - 1$ **do-para** /* Traversing the QSD sequences */
26: **if** $\lfloor \frac{i}{Pr} \rfloor == p$ **then**
27: Traverse the sequence $\acute{q}_i^{p,v}$ to obtain the mode character $\acute{C}_i^{p,v}$ and the set of run-length encoded collection $\acute{E}_i^{p,v}$;
28: $X_i^{p,v} \leftarrow \left\{ \frac{C_i^{p,v}}{n \times N}, \frac{\sum_{j=1}^{n \times N-1} I(\acute{i}_{i,j}^{p,v} - \acute{i}_{i,j-1}^{p,v})}{n \times N - 1}, \frac{\sum_{j=0}^{n \times N-1} (e_{i,j}^{p,v} > 3)}{n \times N}, \frac{Sor(\acute{q}_i^{p,v})}{|\Theta|} \right\}$;
29: Employ $X_i^{p,v}$ and $\{w_0, w_1, w_2, w_3, w_4\}$ according to Eq (5) to obtain the maximum compression gain $y_i^{p,v}$ through the PRPM model;
30: Initialize $j \leftarrow 0$;
31: **while** $j \leq n \times N$ **do**
32: **if** Update $\acute{b}_{i,j}^{p,v}$ and j via $C_i^{p,v}$, $e_{i,j}^{p,v}$, $y_i^{p,v}$, and β using Eq (4) **then** continue;
33: **else if** Update $\acute{b}_{i,j}^{p,v}$ and j based on $C_i^{p,v}$, $e_{i,j}^{p,v}$, $y_i^{p,v}$ and β using Eq (6) **then** continue;
34: **else if** Update $\acute{b}_{i,j}^{p,v}$ and j based on $C_i^{p,v}$, $e_{i,j}^{p,v}$, $y_i^{p,v}$ and β using Eq (7) **then** continue;
35: **else** Update $\acute{b}_{i,j}^{p,v}$ and j based on $C_i^{p,v}$, $e_{i,j}^{p,v}$, $y_i^{p,v}$ and β using Eq (8)
36: **end if**(line-32)
37: **end while**(line-31)
38: The p-th CPU core writes the mode character $C_i^{p,v}$ to the mapping result string $\acute{r}_i^{p,v}$;
39: **for** $j = 0$ to $n \times N - 1$ **do**
40: **if** $\acute{b}_{i,j}^{p,v} \geq 201$ **then** Write $\acute{b}_{i,j}^{p,v}$ and $\acute{q}_{i,j}^{p,v}$ to the string $\acute{r}_i^{p,v}$;
41: **else if** $\acute{b}_{i,j}^{p,v}$!= 'NUL' **then** Write $\acute{b}_{i,j}^{p,v}$ to the string $\acute{r}_i^{p,v}$;
42: **else** continue;
43: **end if**(line-40)
44: **end for**(line-39)
45: **end if**(line-26)
46: **end for**(line-25)
47: **end for**(line-24)
48: **end for**(line-23)

Function : Final compression using ZPAQ and multi-core CPU cluster.

49: Compute $Bl \leftarrow \{bl_0, bl_1, ..., bl_{2\times G-1}\}$ based on the block parameter G and the collection $\acute{R}^v$, where $v = 0, 1$;
50: **for** $nu = 0$ to $Nu - 1$ **do-para** /*Cluster nodes parallel*/
51:     **for** $i = 0$ to $2 \times G - 1$ **do-para** /*Single node multi-core parallel*/
52:         **if** $\lfloor \frac{i}{Nu} \rfloor == nu$ **then**
53:             The nu-th node uses Pr CPU cores use ZPAQ algorithm for parallel compression of bl_i.
54:         **end if**(line-52)
55:     **end for**(line-51)
56: **end for**(line-50)
57: The Pr CPU cores are used in parallel to compress the partition marker collection B using the ZPAQ algorithm;
58: Package the compressed sets B and Bl, along with the parameter n, into a compressed file named **.pqsdc* using the ZPAQ algorithm.
END.

During the execution of Algorithm 1, in steps 1-3, the worst-case time complexity for initializing collections B and F, and parameter $NFactor$ are $O(\frac{m}{Pr})$. The time complexity for parallel filling of the frequency table F in step 4 and parallel computation of the normalization factor in steps 5-11 are $O(\frac{M\times(n-k+1)}{Pr})$. The worst-case time complexity for parallel filling of the set B in steps 12-19 and parallel execution of sequence concatenation in steps 20-21 are $O(\frac{M\times(n-k+1)}{Pr})$. The worst-case time complexity for steps 22-28 is $O\left(\frac{n\times N\times\sum_{v=0}^{1}\frac{B^v}{N}}{Pr}\right)$. The time complexity for calculating the maximum compression gain in step 29 is $O\left(\frac{\sum_{v=0}^{1}\frac{B^v}{N}}{Pr}\right)$. In steps 30-37, the parallel update of result flags $\acute{b}_{i,j}^{p,v}$ has a worst-case time complexity of $O\left(\frac{4\times n\times N\times\sum_{v=0}^{1}\frac{B^v}{N}}{Pr}\right)$. In steps 38-48, the parallel computation of updated $\acute{r}_i^{p,v}$ holds a worst-case time complexity of $O\left(\frac{n\times N\times\sum_{v=0}^{1}\frac{B^v}{N}}{Pr}\right)$. Steps 49-58 utilize the ZPAQ algorithm for futher compression has a worst-case time complexity of $O\left(\frac{z\times(2\times|Bl|)}{Nu\times Pr}\right)$. Here, z represents the average execution time of the ZPAQ algorithm for serially compressing bl_i. Because $\sum_{v=0}^{1}B^v = m$ and k are constants, and $M << m$, the time complexity of PQSDC is $O(max\{\frac{m\times(n-k+1)}{Pr}, \frac{4\times n\times N\times\sum_{v=0}^{1}\frac{B^v}{N}}{Pr}, \frac{z\times(2\times|Bl|)}{Nu\times Pr}\}) = O(max\{\frac{m\times n}{Pr}, \frac{z\times|Bl|}{Nu\times Pr}\})$.

In Algorithm 1, the maximum memory space required for collections Q, $\acute{Q}$, and $\acute{B}^v$ is $O(m\times n)$. The maximum space required for set F is $O(|\Theta|^k)$, the maximum space required for set B^v is $O(\sum_{v=0}^{1}B^v)$. The maximum space overhead of the algorithm ZPAQ is $O(z)$. Because both $|\Theta|$ and k are constants, the spatial complexity of PQSDC is $O(max\{m\times n, z\})$.

2 Supplementary Results and Analysis

2.1 Experimental Datasets

We implement the PQSDC compressor using a combination of C++, MPI, and OpenMP for parallel programming. The performance of PQSDC compressor was evaluated on 27 real-world datasets. These datasets can be obtained from the NCBI database [12] and involve a variety of sequencing platforms and species. The detailed information of the experimental datasets is shown in Table 1.

Table 1: **Detailed information of the experimental datasets.**

ID	Datasets	Platforms	Source	Type	A.Len	Num.Q	Num.Q.C	F.Size
D1	SRR8386204	BGISEQ-500	M.fascicularis	PAIRED	50	39.623	1.981	6.835
D2	SRR8386224	BGISEQ-500	M.fascicularis	PAIRED	50	35.986	1.799	6.204
D3	SRR8386225	BGISEQ-500	M.fascicularis	PAIRED	50	37.682	1.884	6.498
D4	ERR7091256	NextSeq 550	Metagenomic	SINGLE	75	41.351	3.101	9.107
D5	ERR7091267	NextSeq 550	Metagenomic	SINGLE	75	27.258	2.044	5.989
D6	ERR7091268	NextSeq 550	Metagenomic	SINGLE	75	42.914	3.219	9.452
D7	SRR013951	GAIIx	H.sapiens	PAIRED	76	36.425	2.768	9.043
D8	SRR027520	GAIIx	H.sapiens	PAIRED	76	48.493	3.685	12.717
D9	SRR10811412	NextSeq 500	H.sapiens	PAIRED	79	257.518	20.344	19.647
D10	SRR554369	GAIIx	Pseudomonas	PAIRED	100	3.316	0.332	0.871
D11	SRR17794724	MGISEQ-2000RS	M.musculus	PAIRED	100	4.660	0.466	1.249
D12	SRR17794733	MGISEQ-2000RS	M.musculus	PAIRED	100	4.009	0.401	1.074
D13	SRR17794735	MGISEQ-2000RS	M.musculus	PAIRED	100	3.696	0.370	0.990
D14	SRR17794741	MGISEQ-2000RS	M.musculus	PAIRED	100	4.893	0.489	1.312
D15	SRR17794745	MGISEQ-2000RS	M.musculus	PAIRED	100	3.677	0.368	0.985
D16	SRR17794766	MGISEQ-2000RS	M.musculus	PAIRED	100	4.390	0.439	1.176
D17	SRR17794873	MGISEQ-2000RS	M.musculus	PAIRED	100	4.538	0.454	1.216
D18	SRR17794879	MGISEQ-2000RS	M.musculus	PAIRED	100	4.653	0.465	1.247
D19	SRR17794925	MGISEQ-2000RS	M.musculus	PAIRED	100	4.052	0.405	1.086
D20	SRR17794926	MGISEQ-2000RS	M.musculus	PAIRED	100	4.259	0.426	1.141
D21	SRR12175235	NovaSeq 6000	Metagenome	PAIRED	151	17.320	5.231	11.355
D22*	SRR26699593	GridION	L.infantum	SINGLE	4352	1.194	5.197	10.073
D23*	SRR26643265	GridION	E.coli	SINGLE	4686	0.177	0.829	1.605
D24*	SRR26643263	GridION	E.coli	SINGLE	5140	0.163	0.839	1.622
D25*	DRR398855	MinION	E.coli	SINGLE	4660	0.301	1.404	2.700
D26*	DRR398883	MinION	E.coli	SINGLE	8167	0.172	1.403	2.688
D27*	SRR26716641	PacBio RS II	O.curvinotus	SINGLE	8066	0.188	1.513	2.901
**Total**	-	-	-	-	-	632.908	61.857	130.780

A.Len (bp): the average string length of QSD sequences, **Num.Q (Million)**: the total number of QSD sequences, **Num.Q.C (Billion)**: the total number of characters in the QSD sequences; **F.Size (GB)**: the file size of the datasets; "*": the variable-length long-read data.

2.2 Benchmark Algorithms and Parameter Configurations

In our experiment, we compared PQSDC with 4 most advanced compressors, namely CMIC[2], LCQS[3], Qscomp[13], and ZPAQ (method-5) [1]. Among those algorithms, similar to PQSDC, CMIC and LCQS also employ QSD partition and mapping preprocessing strategies. Qscomp utilizes a two-level mapping strategy, and we configure the Qscomp compressor to operate in lossless mode. Since PQSDC utilizes ZPAQ for final compression, it is essential to compare its compression performance with the original ZPAQ algorithm. For these benchmark algorithms, we used the configuration parameters recommended in their manuscripts. The parallel parameter for benchmark algorithms as well as PQSDC^a and PQSDC^b were set to $Pr = 4$ for a fair comparison.

For our parallel algorithm PQSDC, we configured the global parameters based on references [2, 3, 4] and algorithm usage experience. Specifically, we set $k = 4$, $\alpha = 0.150$, $M = 10000$, $N = 100$. To obtain the run-length switch factor and the modeling weights, we randomly selected datasets SRR16552220, SRR11994925, SRR17794717, ERR7091243, and SRR327342 from the NCBI [12] database, comprising a total of 63,510,286 QSD sequences. Through exhaustive calculation, we obtained label values and used multiple linear regression method [9] to fit the parameters. The resulting parameters for the run-length prediction mapping stage take $\beta = 0.340$, $w_0 = 0.945$, $w_1 = -2.128$, $w_2 = 1.065$, $w_3 = 1.198$, and $w_4 = -1.728$. For variable-length long-read sequences, we divide them into short-read with fixed-length 1000 and record the original QSD sequences length additionally.

2.3 Evaluation Metrics

In order to assess the compression effect of the evaluated algorithms, we considered various metrics, including compression ratio, compression robustness, peak memory, time consumption, relative memory consumption, and parallel speedup[3, 7, 5, 14, 15, 4]. The descriptions of evaluation metrics are following.

- **Compression Ratio** (CR, bits/base): The CR is the ratio of the compression size (in bits) of QSD sequences to the total number of QSD characters. It reflects the number of bits needed to store each QSD character. The smaller the CR value, the better the compression efficiency.

- **Coefficient of Variation** (CV, %): The CV is calculated by dividing the standard deviation of compression ratios by the mean compression ratio, then multiplying by 100. A smaller CV value denotes stronger compression robustness and stability.

- **Compression/Decompression Time** (CT/DT, Hours): During compression or decompression process, the CT/DT represents the wall clock time taken by the compressor. Throughput is another evaluation criterion that is related to CT/DT. However, the CT/DT provides a more straightforward insight for algorithm performance.

- **Compression/Decompression Peak Memory** (CPM/DPM, GB): The CPM/DPM is defined as the maximum memory consumption recorded by the computer operation system during the execution of the compression or decompression process. A smaller CPM/DPM denotes that the given compressor is more friendly to memory-limited servers. In computer science, memory consumption is typically evaluated using bytes (B), kilobytes (KB), megabytes (MB), or gigabytes (GB). Here, 1 GB = 1024 MB, 1 MB = 1024 KB, 1 KB = 1024 B, and 1 B = 8 bits.

- **Speedup**: On a single computing node, speedup is defined as the ratio of the serial algorithm's running time to the parallel algorithm's running time. On multiple nodes, speedup represents the ratio of the running time on a single node to the running time on multiple nodes.

- **Relative memory consumption**: (RMO), The RMO is expressed as the ratio of memory consumption between parallel and serial algorithm. On multiple nodes, RMO denotes the ratio of the peak memory on multiple nodes to a single node.

2.4 Compression Performance

2.4.1 Compression Ratio and Robustness

In order to assess the compression ratio and compression robustness of PQSDC and benchmark algorithms, we tested the CR and CR improvement (defined as $\frac{CR_{(benchmark)}-CR_{(ours)}}{CR_{(benchmark)}} \times 100$) of 5 algorithms on the 27 datasets. The experimental results are shown in Table 2.

We first compare PQSDC^a to benchmark algorithms. The results in Table 2 demonstrate that on the short-read datasets (D1∼21), the PQSDC^a achieved the overall best CR values. Compared with ZPAQ, CMIC, LCQS, and Qscomp, PQSDC^a holds average CR improvement percentages (Avg-up†) of 2.458%, 7.209%, 3.053%, and 6.526%, respectively. The weighted average CR improvement percentages (WAvg-up†) are 1.475%, 6.530%, 3.001%, and 8.743%, respectively. On the long-read datasets (D22∼26), compared with CMIC and Qscomp, PQSDC achieved CR improvement of 6.063∼ 9.256% and 6.843∼37.500%, respectively. However, compared with ZPAQ, the CR value of PQSDC^a increased by 0.220∼0.481% on datasets D22∼26. The PQSDC^a performed slightly worse than ZPAQ on variable-length datasets for two main reasons. On the one side, PQSDC requires additional storage to record the length of each long QSD sequence, which increases the overall size of the compressed file. On the other

Table 2: **The compression ratio and compression ratio improvement obtained by PQSDC and benchmark algorithms ZPAQ, CMIC, LCQS, and Qscomp on 27 datasets.**

ID	Compression Ratio (bits/base)						Compression Ratio Improvement (%)			
	PQSDC^b	PQSDC^a	ZPAQ	CMIC	LCQS	Qscomp	ZPAQ	CMIC	LCQS	Qscomp
D1	2.304	**2.303**	2.310	2.511	2.426	2.362	0.303	8.284	5.070	2.498
D2	2.344	**2.343**	2.350	2.554	2.466	2.399	0.298	8.262	4.988	2.334
D3	2.317	**2.316**	2.327	2.523	2.440	2.374	0.473	8.205	5.082	2.443
D4	0.295	**0.283**	0.288	0.297	0.316	0.347	1.736	4.714	10.443	18.444
D5	0.320	**0.308**	0.314	0.323	0.346	0.377	1.911	4.644	10.983	18.302
D6	0.327	**0.315**	0.321	0.330	0.354	0.386	1.869	4.545	11.017	18.394
D7	3.898	**3.894**	3.935	4.088	3.933	4.468	1.042	4.746	0.992	12.847
D8	3.072	**3.069**	3.101	3.244	3.089	3.354	1.032	5.395	0.647	8.497
D9	0.012	**0.001**	0.002	0.004	**0.001**	0.004	50.000	75.000	0.000	75.000
D10	2.395	2.390	**2.339**	2.532	2.390	2.958	-2.180	5.608	0.000	19.202
D11	2.195	**2.193**	2.284	2.383	2.260	2.269	3.984	7.973	2.965	3.349
D12	2.148	**2.147**	2.243	2.330	2.219	2.215	4.280	7.854	3.245	3.070
D13	2.273	**2.271**	2.367	2.463	2.340	2.379	4.056	7.795	2.949	4.540
D14	2.294	**2.293**	2.389	2.499	2.365	2.373	4.018	8.243	3.044	3.371
D15	2.190	**2.188**	2.284	2.378	2.259	2.269	4.203	7.990	3.143	3.570
D16	2.172	**2.171**	2.268	2.360	2.243	2.250	4.277	8.008	3.210	3.511
D17	2.204	**2.203**	2.301	2.395	2.275	2.282	4.259	8.017	3.165	3.462
D18	2.337	**2.336**	2.430	2.527	2.405	2.453	3.868	7.558	2.869	4.770
D19	2.157	**2.155**	2.252	2.338	2.227	2.226	4.307	7.827	3.233	3.190
D20	2.324	**2.323**	2.414	2.521	2.388	2.421	3.770	7.854	2.722	4.048
D21	0.499	**0.496**	0.502	0.521	0.513	0.633	1.195	4.798	3.314	21.643
D22	3.676	3.675	**3.664**	4.004	###	4.389	-0.300	8.217	###	16.268
D23	3.643	3.641	**3.633**	3.876	###	4.387	-0.220	6.063	###	17.005
D24	3.642	3.640	**3.632**	3.875	###	4.386	-0.220	6.065	###	17.009
D25	4.589	4.594	**4.572**	5.049	###	4.932	-0.481	9.012	###	6.853
D26	4.589	4.588	**4.568**	5.056	###	4.925	-0.438	9.256	###	6.843
D27	0.006	**0.005**	**0.005**	**0.005**	###	0.008	0.000	0.000	###	37.500
**Avg-up**†	-	-	-	-	-	-	2.458	7.209	3.053	6.526
**Wavg-up**†	-	-	-	-	-	-	1.475	6.530	3.001	8.743
**Avg-up**	-	-	-	-	-	-	1.591	7.478	32.638	8.655
**WAvg-up**	-	-	-	-	-	-	0.693	7.254	37.221	10.779
**SSP**	-	-	-	-	-	-	0.718	7.250	37.208	10.799
**CV**	58.768	58.970	**57.971**	59.158	82.001	60.830	-	-	-	-

PQSDC^a: CPU multi-core acceleration ($Pr = 4$); **PQSDC^b**: CPU clusters acceleration ($Pr = 4$, $G = 3$, $Nu = 3$); ###: failed or runtime over 72 hours; **Avg-up (%)**: the average percentage improvement in CR of PQSDC^a compared to the benchmark algorithm (For the compressor that failed to run, we set CR = 8 bits/base); **WAvg-up (%)**: weighted average percentage improvement in CR, where the weights are based on the file size proportions; **SSP (%)**: storage saving percentage; "†": exclude datasets D22 to D27 from the calculation. The best result in the table is marked with boldface.

hand, PQSDC fitted the weight parameters $\{w_0, w_1, w_2, w_3, w_4\}$ on short-read data, resulting in inferior generalization performance of CR on the long-read datasets. Considering the overall performance on the 27 datasets, our PQSDC^a algorithm, accelerated by multi-core CPUs, exhibits an improvement of 1.591∼32.638% and 0.718∼37.208% in Avg-up and WAvg-up compared with the four benchmark methods, and gains additional storage saving percentage (SSP) of 0.718%, 7.250%, 37.208%, and 10.799%, respectively. Although PQSDC shows a relatively slight improvement in CR and SSP compared with ZPAQ, it significantly outperforms ZPAQ regarding time and memory consumption (detailed in the following subsection).

Although the overall CR of PQSDC^b is slightly inferior to PQSDC^a (due to introducing the chunk parameter G for CPU cluster parallel computing), the overall performance still outperforms the benchmark algorithms employed in the experiments. Additionally, on compression robustness (CV), both PQSDC^a and PQSDC^b compressors acquired CV values close to ZPAQ, superior to CMIC, LCQS, and Qscomp. This result shows that CMIC, LCQS, and Qscomp are more sensitive to the QSD probability distribution of the datasets to be compressed, while our PQSDC is more robust and stable.

In datasets D9 and D27, we observed two extreme CR value groups. Particularly on D9, PQSDC^a showed a significant improvement in CR compared with ZPAQ, CMIC, and Qscomp, with CR improve-

ment of 50%, 75%, and 75%, respectively. We attribute this phenomenon to "continuous repetitive patterns" in the QSD collections. Since PQSDC employs run-length encoding in its PRPM model, it eliminates redundancy QSD characters on such datasets more effectively. Another interesting result was also observed in D9, where the CR of cluster-accelerated PQSDC^b was 12 times higher than PQSDC^a. This phenomenon may be attributed to the improper segmentation of the block parameter G in PQSDC^b.

2.4.2 Time Consumption

Table 3 shows the CT and DT obtained by CPU multi-core accelerated PQSDC^a, CPU cluster accelerated PQSDC^b, and 4 benchmark algorithms on 27 datasets. The PQSDC^b algorithm has the lowest overall CT and DT cost. The CT cost of PQSDC^b, LCQS, CMIC, PQSDC^a, ZPAQ, and Qscomp get bigger successively. Moreover, the overall DT cost of PQSDC^b, LCQS, PQSDC^a, CMIC, ZPAQ, and Qscomp get bigger successively. The above 6 methods have total compression and decompression time costs (CT+DT, Hours) of 4.015, 6.056, 15.074, 6.889, 4.634, and 22.661 on the datasets D1$\sim$21, respectively. The PQSDC^b achieved reductions of 33.702%, 73.365%, 41.719%, 13.358%, and 82.282% in CT+DT costs, compared with PQSDC^a, ZPAQ, CMIC, LCQS, and Qscomp, respectively. The PQSDC^a, without CPU cluster acceleration, still exhibited lower total time consumptions, ranking second only to LCQS. The PQSDC^a reduced total time costs (CT+DT) by 12.092%, 59.825%, and 73.276%, compared with CMIC, ZPAQ, and Qscomp, respectively. Those results show that the PQSDC algorithm has a competitive advantage regarding time performance, especially the PQSDC^b which is accelerated with CPU clusters.

Table 3: **The compression and decompression time obtained by the PQSDC and benchmark algorithms ZPAQ, CMIC, LCQS, and Qscomp on 27 datasets.**

ID	Compression Time (Hours)						Deompression Time (Hours)					
	PQSDC^b	PQSDC^a	ZPAQ	CMIC	LCQS	Qscomp	PQSDC^b	PQSDC^a	ZPAQ	CMIC	LCQS	Qscomp
D1	**0.140**	0.241	0.331	0.235	0.176	0.436	**0.107**	0.188	0.338	0.236	0.168	0.441
D2	**0.136**	0.228	0.292	0.298	0.201	0.445	**0.106**	0.174	0.296	0.322	0.195	0.398
D3	**0.250**	0.298	0.375	0.297	0.209	0.431	**0.194**	0.242	0.377	0.301	0.200	0.407
D4	**0.098**	0.098	0.401	0.110	0.057	0.631	**0.086**	0.055	0.404	0.112	0.055	0.635
D5	0.055	0.079	0.275	0.067	**0.041**	0.423	0.051	0.048	0.274	0.068	**0.038**	0.427
D6	0.064	0.120	0.424	0.115	**0.062**	0.653	**0.056**	0.077	0.424	0.114	0.061	0.657
D7	**0.338**	0.606	0.435	0.513	0.456	0.682	**0.195**	0.400	0.441	0.518	0.422	0.804
D8	**0.310**	0.601	0.527	0.670	0.471	0.839	**0.204**	0.471	0.535	0.682	0.444	0.842
D9	**0.098**	0.336	2.885	0.167	0.135	4.449	**0.069**	0.069	2.869	0.163	0.123	4.477
D10	0.050	0.059	0.077	0.061	**0.031**	0.079	0.048	0.049	0.077	0.061	**0.034**	0.078
D11	0.068	0.060	0.079	0.059	**0.041**	0.118	0.061	0.050	0.079	0.058	**0.039**	0.119
D12	0.052	0.063	0.082	0.061	**0.036**	0.122	0.051	0.053	0.083	0.062	**0.036**	0.123
D13	0.053	0.065	0.075	0.061	**0.034**	0.087	0.047	0.055	0.079	0.064	**0.034**	0.089
D14	0.071	0.064	0.074	0.060	**0.046**	0.121	0.062	0.049	0.074	0.059	**0.043**	0.121
D15	0.052	0.065	0.075	0.062	**0.035**	0.094	0.047	0.053	0.078	0.062	**0.037**	0.094
D16	0.060	0.068	0.077	0.056	**0.037**	0.123	0.051	0.049	0.077	0.059	**0.036**	0.124
D17	0.063	0.071	0.077	0.065	**0.041**	0.125	0.057	0.058	0.081	0.067	**0.038**	0.126
D18	0.066	0.069	0.078	0.062	**0.045**	0.118	0.059	0.055	0.078	0.065	**0.038**	0.122
D19	0.056	0.069	0.084	0.066	**0.037**	0.107	0.051	0.058	0.086	0.066	**0.037**	0.107
D20	0.061	0.067	0.083	0.064	**0.038**	0.109	0.054	0.054	0.084	0.063	**0.040**	0.109
D21	**0.119**	0.250	0.719	0.266	0.141	1.081	**0.100**	0.174	0.719	0.271	0.142	1.089
D22	**0.518**	2.101	0.937	1.393	###	1.282	**0.322**	0.992	1.029	1.396	###	1.296
D23	**0.107**	0.338	0.147	0.187	###	0.194	**0.063**	0.154	0.152	0.189	###	0.194
D24	**0.106**	0.332	0.155	0.198	###	0.193	**0.069**	0.156	0.157	0.200	###	0.196
D25	**0.225**	0.770	0.263	0.299	###	0.324	**0.126**	0.259	0.269	0.301	###	0.329
D26	**0.230**	0.746	0.259	0.299	###	0.323	**0.125**	0.259	0.269	0.303	###	0.326
D27	**0.013**	0.028	0.169	0.021	###	0.263	**0.012**	0.019	0.166	0.020	###	0.264
**Total**	**2.259**	3.575	7.523	3.414	2.371	11.272	**1.756**	2.479	7.551	3.475	2.260	11.389

Notes. LCQS took more than 72 hours on datasets D22$\sim$27, the total time only includes calculations for D1$\sim$21. The best result in the table is marked with boldface.

We found that PQSDC^b merely reduced total CT and DT percentages compared with PQSDC^a by 36.811% and 29.156%, respectively. This phenomenon is caused by the insufficient parallelism of

PQSDC^b and the relatively small single-file size. For example, considering datasets D10∼20, the file size limits the time acceleration by CPU clusters more significantly.

Another interesting phenomenon is that the compression time of CMIC, LCQS, and PQSDC does not increase in direct proportion to the dataset size. For example, in D1∼D21, the top-3 datasets in terms of file size are D9, D21, and D8. The ZPAQ and Qscomp compressors have the highest time consumption on the D9 dataset. However, CMIC, LCQS, and PQSDC all have shorter compression times on the dataset D9 compared to D8. This is because factors affecting compression time include not only the size of the dataset but also the probability distribution of QSD. When there are many redundant QSD sequences in the dataset, it benefits PQSDC, CMIC, and LCQS. These compressors utilize mapping strategies to leverage this redundancy, reducing the size of the dataset to be compressed. When ZPAQ is used for post-processing, it helps reduce the compression time overhead.

2.4.3 Peak Memory Consumption

Table 4 presents the peak memory consumption CPM and DPM of PQSDC^a, PQSDC^b, ZPAQ, CMIC, LCQS, and Qscomp.

Table 4: **The compression and decompression peak memory obtained by the PQSDC and benchmark algorithms ZPAQ, CMIC, LCQS, and Qscomp on 27 datasets.**

ID	Compression Peak Memory (GB)						Deompression Peak Memory (GB)					
	PQSDC^b	PQSDC^a	ZPAQ	CMIC	LCQS	Qscomp	PQSDC^b	PQSDC^a	ZPAQ	CMIC	LCQS	Qscomp
D1	**2.464**	3.738	3.747	3.880	8.393	3.499	**2.342**	3.245	3.358	3.316	5.481	3.261
D2	**2.438**	3.710	3.750	3.886	8.215	3.531	**2.319**	3.312	3.350	3.316	5.756	3.281
D3	**2.481**	3.698	3.742	3.882	8.435	3.528	**2.358**	3.260	3.345	3.316	5.341	3.248
D4	**1.126**	2.922	3.145	3.425	3.991	3.517	**0.696**	2.803	2.995	3.269	10.604	3.268
D5	**0.752**	2.216	3.125	3.207	3.873	3.530	**0.728**	2.160	2.904	3.086	7.251	3.304
D6	**1.397**	2.965	3.155	3.469	4.104	3.464	**1.339**	2.812	2.906	3.270	11.105	3.280
D7	3.831	3.817	3.806	3.862	8.020	**3.750**	3.308	**3.221**	3.284	3.314	5.559	3.257
D8	3.877	3.820	3.738	3.865	14.813	**3.616**	3.316	3.315	3.290	3.359	6.985	**3.263**
D9	**0.706**	0.707	2.552	3.320	2.219	2.932	0.658	**0.656**	2.027	3.066	43.167	2.441
D10	**0.797**	1.630	3.511	2.619	3.916	3.456	**0.776**	1.565	3.378	2.520	2.989	3.177
D11	**0.814**	1.742	3.587	2.919	5.720	3.512	**0.795**	1.695	3.317	2.752	3.637	3.295
D12	**0.779**	1.601	3.486	2.558	4.151	3.402	**0.761**	1.548	3.289	2.445	3.018	3.168
D13	**0.508**	1.312	3.451	2.510	3.885	3.478	**0.487**	1.244	3.278	2.393	2.993	3.105
D14	**0.815**	1.729	3.571	3.332	5.189	3.414	**0.791**	1.655	3.279	3.165	3.270	3.018
D15	**0.504**	1.309	3.452	2.484	3.793	3.492	**0.487**	1.248	3.282	2.370	3.000	3.120
D16	**0.793**	1.634	3.522	2.636	4.427	3.511	**0.771**	1.575	3.280	2.489	3.098	3.285
D17	**0.799**	1.640	3.535	2.862	4.700	3.507	**0.776**	1.577	3.276	2.689	3.283	3.266
D18	**0.805**	1.674	3.547	2.953	4.789	3.507	**0.780**	1.596	3.279	2.784	3.349	3.267
D19	**0.790**	1.626	3.492	2.569	4.071	3.413	**0.770**	1.565	3.274	2.454	3.013	3.030
D20	**0.802**	1.661	3.572	2.633	4.533	3.403	**0.780**	1.587	3.315	2.490	3.367	3.011
D21	**3.017**	3.220	3.182	3.658	5.030	3.616	2.862	**2.801**	2.839	3.271	14.939	3.204
D22	3.828	3.816	**3.782**	3.822	###	3.680	3.316	**3.301**	3.298	3.319	###	3.292
D23	**2.901**	3.801	3.770	3.813	###	3.587	**2.736**	3.285	3.291	3.314	###	3.290
D24	**2.842**	3.790	3.772	3.814	###	3.584	**2.669**	3.277	3.290	3.316	###	3.298
D25	**3.601**	3.843	3.833	3.906	###	3.687	3.315	**3.261**	3.287	3.317	###	3.291
D26	**3.600**	3.843	3.833	3.903	###	3.796	3.316	**3.265**	3.283	3.315	###	3.295
D27	**0.257**	1.919	2.150	0.437	###	2.561	**0.253**	0.428	2.449	0.436	###	2.422
**Avg**	**1.753**	2.303	3.460	3.168	5.537	3.480	**1.611**	2.116	3.169	2.911	7.200	3.169

Notes. LCQS took more than 72 hours on D22∼27, the average peak memory only includes calculations for D1∼21. The best result in the table is marked with boldface.

The results presented indicate that our PQSDC^a and PQSDC^b methods have the lowest overall CPM and DPM on the tested datasets. PQSDC^b requires smaller memory than PQSDC^a, ZPAQ, CMIC, LCQS, and Qscomp, with only 68.302%, 50.665%, 55.335%, 31.660%, and 50.374% of their average CPM consumption, respectively. Similarly, PQSDC^b shows lower memory requirements in decompression as well, with only 78.497%, 52.414%, 55.342%, 22.375%, and 50.836% of their average CPM consumption, respectively. These results demonstrate that PQSDC can effectively run on memory-limited computer

systems, thanks to the PSPM and PRPM optimization techniques employed by PQSDC.

2.5 Algorithm Ablation Experiments

In order to evaluate the effect of the PSPM (Parallel Sequences Partition Model), PRPM (Parallel Run-length Prediction Mapping Model), and ZPAQ on the PQSDC compressor, we tested the following ablation experimental combination: PSPM+ZPAQ, PRPM+ZPAQ, Pure ZPAQ, and PSPM+PRPM+ZPAQ (equivalent to PQSDC[a]). We measured their average compression ratio (Avg-CR), weighted average compression ratio (WAvg-CR), compression robustness (CV), total compression time (Total-CT), total decompression time (Total-DT), average compression peak memory (Avg-CPM), and average decompression peak memory (Avg-DPM) on 27 datasets. The experimental results are shown in Table 5.

Table 5: **Results of the ablation experiments on 27 datasets using the proposed PQSDC compressor (*Pr*=4).**

Modules	Avg-CR (bits/base)	WAvg-CR (bits/base)	CV (%)	Total-CT (Hours)	Total-DT (Hours)	Avg-CPM (GB)	Avg-DPM (GB)
PSPM+ZPAQ	2.238	1.429	58.749	7.582	6.715	3.247	3.002
PRPM+ZPAQ	2.238	1.431	58.745	**4.020**	4.125	3.192	2.843
Pure ZPAQ	2.458	1.475	**57.971**	7.523	7.551	3.460	3.169
PSPM+PRPM+ZPAQ	**2.228**	**1.425**	58.979	4.821	**3.992**	**2.576**	**2.269**

The best result in the table is marked with boldface.

The results in Table 5 show that using only the combination of PRPM+ZPAQ exhibits notable performance improvements in terms of Avg-CR, Wavg-CR, Total-CT, Total-DT, Avg-CPM, Avg-DPM compared with Pure ZPAQ. This indicates that the innovative run-length prediction mapping method of PRPM effectively reduces redundancy in QSD data. The combination of PRPM+ZPAQ has advantages over Pure ZPAQ in terms of time and memory usage. The combination of PSPM+ZPAQ also improves performance in Avg-CR, WAvg-CR, and Total-DT, compared with Pure ZPAQ, while the improvement is smaller than that of the PRPM+ZPAQ combination. However, incorporating the PSPM model on top of PRPM+ZPAQ leads to even more significant overall performance improvements in PQSDC, especially regarding Avg-CR, WAvg-CR, Total-DT, Avg-CPM and Avg-DPM.

Our ablation experiments have demonstrated that combining PSPM and PRPM is indispensable for PQSDC. By leveraging the strengths of both modules, PQSDC not only improves the compression ratio but also ensures overall advantages in terms of time cost and peak memory consumption.

2.6 Parallel Performance

In this subsection, we first tested the impact of multi-core acceleration on the compression performance of PQSDC. Then, we evaluated the scalability of the PQSDC algorithm as data size and the number of CPU cores changed. Similarly, we also tested the influence of parallel scalability and compression performance when PQSDC enabled multiple CPU clusters.

2.6.1 Multi-core CPU Parallel Acceleration

To discover the impact of PQSDC multi-core parallel acceleration on compression performance, the experimental evaluation of the PQSDC algorithm was conducted on the SRR027550 dataset using a single node ($Nu = 1$) with varying numbers of CPU cores (Pr). The results, including compression ratio, time, peak memory usage, parallel speedup, and relative memory overhead, are presented in Table 6. Table 6 experimental results demonstrate that parallelizing the PQSDC algorithm does not affect the CR due to the high independence among the computed data. Regarding CT and DT, the peak parallel speedup was achieved when the number of cores reached 28. Notably, the parallel acceleration

Table 6: **Experimental results obtained by running the parallel algorithm PQSDC with different numbers of CPU cores on the SRR027520 dataset. (*Nu*=1, *G*=1)**

Pr	PQSDC Compression					PQSDC Decompression			
	CR (bits/base)	CT (S)	Speedup (Ratio)	CPM (MB)	RMO (Ratio)	DT (S)	Speedup (Ratio)	DPM (MB)	RMO (Ratio)
1	3.069	7450.001	1.000	957.543	1.000	5600.001	1.000	850.879	1.000
4	3.069	2401.086	3.103	3978.738	4.155	1675.038	3.343	3395.070	3.990
8	3.069	1455.041	5.120	7983.023	8.337	999.051	5.605	6774.074	7.961
12	3.069	1164.060	6.400	11961.078	12.491	852.024	6.573	10142.324	11.920
16	3.069	954.024	7.809	14831.527	15.489	693.047	8.080	13539.289	15.912
20	3.069	888.011	8.390	18107.707	18.911	670.027	8.358	16903.875	19.866
24	3.069	862.079	8.642	21362.672	22.310	663.050	8.446	20198.352	23.738
28	3.069	734.079	10.149	22808.598	23.820	523.037	10.707	21768.387	25.583

effect of the PQSDC on the SRR027520 dataset outperforms the logarithmic speedup[5], exhibiting a predominantly linear growth trend. Regarding CPM and DPM, parallelizing the PQSDC using the ZPAQ compressor and shared memory parallel programming model requires additional memory space to store intermediate calculation results. As a result, the RMO of CPM and DPM exhibit a linear positive correlation with the number of enabled CPU cores (*Pr*).

In order to evaluate the scalability of the PQSDC, experiments were conducted on six randomly selected datasets ranging in size from 495 MB to 5249 MB. The results of the parallel scalability evaluation are shown in Fig 4.

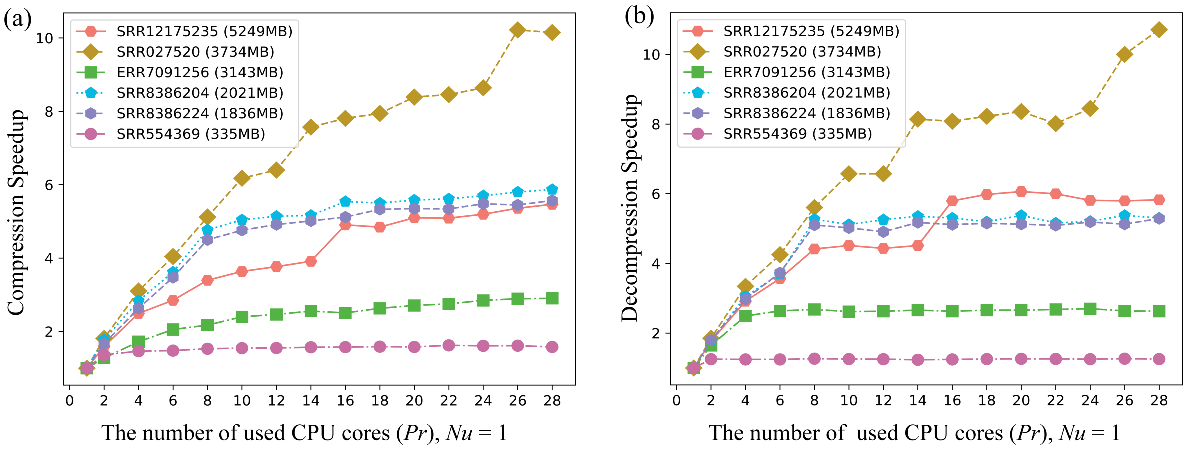

Figure 4: **The parallel speedup obtained by running the PQSDC with different numbers of CPU cores on six datasets.** (a) CPU multi-core parallel compression speedup. (c) CPU multi-core parallel decompression speedup.

The experimental results in Fig 4 demonstrate that the PQSDC algorithm shows an enhanced trend in speedup as the number of CPU cores increases. As the dataset size increases, the parallel acceleration effect becomes more pronounced. In Fig 4(a), when CPU cores were set as 28, 27, 27, 26, 24, and 14, the PQSDC reached peak parallel compression speedup, respectively. Similarly, in Fig 4(b), at cores 22, 28, 24, 20, 20, and 12, the PQSDC achieved peak parallel decompression speedup, respectively.

2.6.2 Multi-node CPU Cluster Parallel Acceleration

The PQSDC applies a data block-cycle-parallel strategy to the ZPAQ compressor for compression and decompression of processed data. In order to evaluate the influence of CPU cluster parallel acceleration on algorithm performance, this section first gives the compression ratio, time, parallel speedup, peak

memory, and relative memory overhead obtained when the PQSDC is run on the SRR027520 dataset with different numbers of CPU cluster nodes (Nu). The experimental results are shown in Table 7.

Table 7: **Experimental results obtained by running the parallel algorithm PQSDC with different numbers of CPU cluster nodes on the SRR027520 dataset (Pr=4, G=6).**

	PQSDC Compression					PQSDC Decompression			
Nu	**CR (bits/base)**	**CT (S)**	**Speedup (Ratio)**	**CPM (MB)**	**RMO (Ratio)**	**DT (S)**	**Speedup (Ratio)**	**DPM (MB)**	**RMO (Ratio)**
1	3.073	3078.000	1.000	3586.016	1.000	2569.000	1.000	3394.441	1.000
2	3.073	1917.000	1.605	3586.820	1.000	1366.000	1.882	3393.156	0.999
3	3.073	1540.000	1.999	3589.844	1.001	1077.000	2.384	3394.443	1.000
4	3.073	1364.000	2.257	3585.996	0.999	768.000	3.345	3390.000	0.999
5	3.073	1262.000	2.437	3589.996	1.001	584.000	4.396	3394.781	1.000
6	3.073	1086.000	2.833	3588.504	1.001	514.000	4.998	3397.629	1.004
7	3.073	1091.000	2.820	3585.973	0.999	513.000	5.006	3393.270	0.999

The results in Table 7 indicate that when the block parameter G is fixed, the variation of Nu value did not affect the CR and RMO (both CPM and DPM). Regarding speedup of CT and DT, the SRR027520 dataset reaches the peak compression and decompression speedup when the number of nodes takes 6 and 7, with peak parallel speedup of 2.833 and 5.006, respectively.

In order to determine the scalability of the CPU cluster parallel algorithm PQSDC, our experiment subsequently evaluated the cluster parallel scalability. The experimental results are shown in Fig 5.

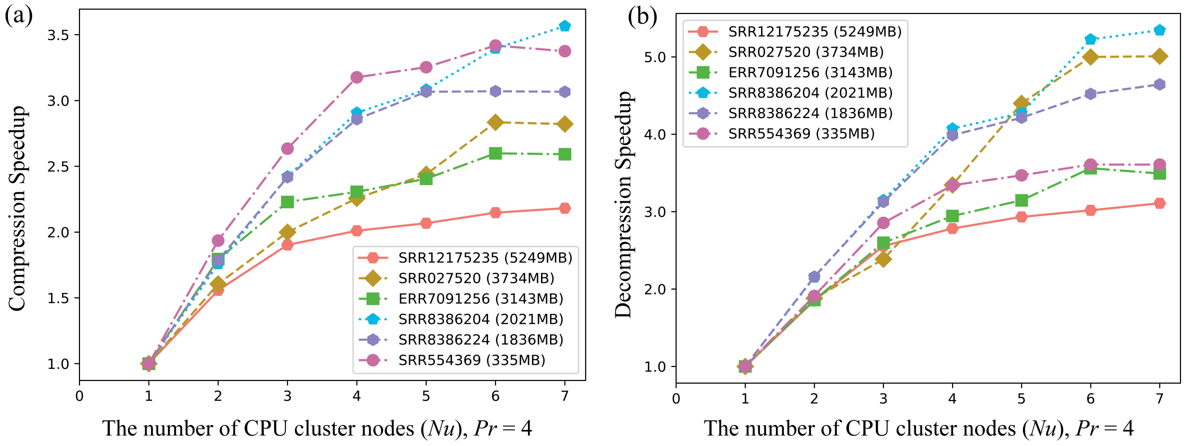

Figure 5: **The parallel speedup obtained by running the PQSDC with different numbers of CPU cluster nodes on six datasets.** (a) CPU cluster parallel compression speedup. (c) CPU cluster parallel decompression speedup.

The experimental results shown in Fig 5 indicate that the CPU cluster parallel algorithm PQSDC achieves a maximum compression speedup of 2∼3.5 times and a maximum decompression speedup of 3∼5 times on six datasets. The acceleration effect stabilizes when the number of cluster nodes (Nu) is between 5 and 7. When the number of cluster nodes (Nu) ranges from 5 to 7, the speedup of PQSDC tends to stabilize. This phenomenon can be attributed to two aspects: (i) The block parameter G limits PQSDC's computational load balance when block data cannot be evenly distributed among each CPU cluster node. (ii) As the number of CPU cluster nodes increases, the PSPM and PRPM models take up a more significant proportion of the total runtime. These stages are complex and challenging to speed up using CPU clusters.

References

[1] Matt Mahoney. *Incremental Journaling Backup Utility and Archiver.* 2016. URL: `https://mattmahoney.net/dc/zpaq.html`.

[2] Hansen Chen et al. "CMIC: an efficient quality score compressor with random access functionality". In: *BMC bioinformatics* 23.1 (2022), pp. 1–17.

[3] Jiabing Fu, Bixin Ke, and Shoubin Dong. "LCQS: an efficient lossless compression tool of quality scores with random access functionality". In: *BMC bioinformatics* 21.1 (2020), pp. 1–12.

[4] Cheng Zhong and Hui Sun. "Parallel algorithm for sensitive sequence recognition from long-read genome data with high error rate". In: *Journal on Communications* 44.160-171 (2023). ISSN: 1000-436X.

[5] Peter Pacheco. *An introduction to parallel programming.* Elsevier, 2011.

[6] Nicholas Wilt. *The cuda handbook: A comprehensive guide to gpu programming.* Pearson Education, 2013.

[7] Greg Malysa et al. "QVZ: lossy compression of quality values". In: *Bioinformatics* 31.19 (2015), pp. 3122–3129.

[8] Khalid Sayood. *Introduction to data compression.* Morgan Kaufmann, 2017.

[9] Dastan Maulud and Adnan M Abdulazeez. "A review on linear regression comprehensive in machine learning". In: *Journal of Applied Science and Technology Trends* 1.4 (2020), pp. 140–147.

[10] Claude Elwood Shannon. "A mathematical theory of communication". In: *The Bell system technical journal* 27.3 (1948), pp. 379–423.

[11] Dohyeon Lee and Giltae Song. "FastqCLS: a FASTQ compressor for long-read sequencing via read reordering using a novel scoring model". In: *Bioinformatics* 38.2 (2022), pp. 351–356.

[12] Lewis Y Geer et al. "The NCBI biosystems database". In: *Nucleic acids research* 38.suppl_1 (2010), pp. D492–D496.

[13] Jan Voges et al. "A two-level scheme for quality score compression". In: *Journal of Computational Biology* 25.10 (2018), pp. 1141–1151.

[14] Hui Sun et al. "PMFFRC: a large-scale genomic short reads compression optimizer via memory modeling and redundant clustering". In: *BMC bioinformatics* 24.1 (2023), p. 454.

[15] Yuting Xing et al. "GTZ: a fast compression and cloud transmission tool optimized for FASTQ files". In: *BMC bioinformatics* 18.16 (2017), pp. 233–242.
